# Why does the latest pharmacovigilance data not reflect clinical experience with dydrogesterone?

**DOI:** 10.1093/hropen/hoaf030

**Published:** 2025-05-26

**Authors:** Emre Pabuccu, Angela Aguilar, Roberto de Azevedo Antunes, Eran Gefen, Lee P Shulman

**Affiliations:** Ufuk University School of Medicine, Dr. Rıdvan Ege Hospital, Ankara, Turkey; Centrum Clinic Assisted Reproduction Center, Ankara, Turkey; Reproductive Medicine, Obstetrics and Gynecology, University of the Philippines, Manila, Philippines; Fertipraxis Centro de Reprodução Humana, Rio de Janeiro, Brazil; Gynecology Departament, Clementino Fraga Filho Hospital, Federal University of Rio de Janeiro, Rio de Janeiro, Brazil; Global Medical Affairs & PV, Abbott Product Operations, Allschwil, Switzerland; Division of Clinical Genetics, Department of Obstetrics & Gynecology, Feinberg School of Medicine, Northwestern University, Chicago, IL, USA

Dear Editor,

We read with interest the recent article by Henry *et al.* (2025) and the invited commentary by Parazzini *et al.* (2025) that were recently published in *Human Reproduction Open*. We agree that pharmacovigilance (PV) data are important in highlighting potential real-world safety issues. However, PV is a specialized area that requires contextualization, especially regarding factors that may confound or introduce bias. Therefore, the purpose of this Letter is to offer a clinically focused and rational interpretation of the PV data to reassure clinicians who use dydrogesterone in their practice.

The reporting rate of birth defects should be seen in the context of the general ART population, in which a global incidence rate of ∼6% has been reported (Kang *et al.*, 2023). However, the authors report a rate of 0.7%, suggesting serious under-reporting within the database. Although study limitations are described, their significance is underemphasized.

Historically, the use of dydrogesterone was primarily focused outside of Europe and North America in different indications. First approved for ART in Europe in 2017, dydrogesterone is now available in six EU countries. It is well-known that a newly approved drug often leads to an increase in PV case reports, and most such reports occurred between 2011 and 2020, suggesting a high likelihood of temporal bias. Since 2015, a small number of publications have raised concerns regarding a potential association between dydrogesterone and birth defects; however, the most prominent among them was later retracted due to methodological flaws and concerns over study design (https://link.springer.com/article/10.1007/s40261-019-00862-w). These factors, combined with the high maternal tolerability of dydrogesterone, suggest a potential bias in the reporting of adverse events (AEs) related to birth defects but not maternal effects—despite similar rates of maternal AEs between dydrogesterone and progesterone (Griesinger *et al.*, 2020).

The skew towards reporting more birth defects associated with dydrogesterone due to these biases explains the high reporting odds ratio (ROR). In case–non-case methodological studies, a small number of AEs beyond the event of interest (‘non-cases’) drives the ROR upwards (Parazzini *et al.*, 2025). This methodological flaw arises due to the narrower ratio between cases: non-cases for dydrogesterone (60:85 [1:1.4]) compared with progesterone (141:1081 [1:7.7]). The supplementary data from Henry *et al.* (2025) suggest that the tolerability of dydrogesterone for mothers compares favourably with progesterone. Finally, selection bias cannot be estimated as miscarriage history was not provided, despite the association between previous miscarriage and increased risk of birth defects (Campaña *et al.*, 2017).

A meta-analysis by Katalinic *et al.* (2024) provided the most robust evidence to date regarding the safety of dydrogesterone in newborns. However, it also highlighted the overall paucity of high-quality data, with most available evidence derived from small, observational cohort studies. Key limitations included a lack of standardized outcome measures, retrospective data collection, and heterogeneity in study design, which should be explicitly acknowledged to allow a balanced interpretation of the results. Using the ROBINS-I tool (Sterne *et al.*, 2016), Zaqout *et al.* (2015)—a retrospective cohort study investigating the association between dydrogesterone exposure in early pregnancy and congenital heart defects—was rated at critical risk of bias, mainly due to serious limitations in verifying dydrogesterone intake. Similarly, the recent publication by Li *et al.* (2024), which reported an unexpectedly high number of birth defect cases linked to dydrogesterone in a PV database, lacks detailed exposure data and population-level context, raising concerns about data interpretation and potential reporting bias. There is also widespread literature documenting the lack of biological plausibility for any teratogenic or androgenic effects of dydrogesterone (Katalinic *et al.*, 2022). Furthermore, the reporting of PV data should follow the READUS-PV guidance (www.readus-statement.org), including a review of all relevant evidence already accrued. However, although a letter to the Editor (Quadros *et al.*, 2024) criticizing the conclusions of this most recent meta-analysis is cited in Henry *et al.* (2025), the corresponding reply that highlights the authors’ likely misunderstanding of the methodological principles (Katalinic, 2024) was not.

Over the past 65 years, >147 million women have been treated with dydrogesterone, with >20 million successful pregnancies (Katalinic *et al.*, 2022). While we recognise the role of PV in detecting early safety signals, its decontextualised use may deny patients access to an important medication. Dydrogesterone offers an oral option that is important in many regions for cultural and ethnic reasons. We advocate for a more objective and responsible interpretation of the Henry *et al.* (2025) study that clarifies and contextualizes the PV data.
